# Epidemiology and Genetic Characterization of Porcine Parvovirus 7 Recovered from Swine in Hunan, China

**DOI:** 10.3390/ani14152222

**Published:** 2024-07-31

**Authors:** Dongliang Wang, Qing He, Naidong Wang, Jinhui Mai

**Affiliations:** 1 College of Biology, Hunan University, Changsha 410082, China; dongliangwang@hnu.edu.cn; 2 Hunan Provincial Key Laboratory of Protein Engineering in Animal Vaccines, Laboratory of Functional Proteomics (LFP), Research Center of Reverse Vaccinology (RCRV), College of Veterinary Medicine, Hunan Agricultural University, Changsha 410128, China; qinghe@stu.hunau.edu.cn

**Keywords:** PPV7, cap, epidemiology, genetic characterization

## Abstract

**Simple Summary:**

Porcine parvovirus 7 (PPV7) infection or co-infections with other pathogens have been documented in aborted pig fetuses and in sows that experienced reproductive failure, which has led to concern about the viral infection. Therefore, it is important to investigate the prevalence and genetic characterization of PPV7. In this study, we reported the prevalence and genetic characteristics of PPV7 circulating in swine in Hunan, China. These results will be of interest to those working in virus evolutionary dynamics analysis and would be helpful to provide the impetus and basis to further elucidate the pathogenesis of PPV7.

**Abstract:**

Porcine parvovirus 7 (PPV7) was first discovered in swine in 2016, and PPV7 infection has been detected in aborted pig fetuses and in sows that experienced reproductive failure. The objective of this study was to report the prevalence and genetic characterization of PPV7 in Hunan, China. Seventy of the four hundred and twenty-two (16.6%) serum, semen, and tissue samples collected from pigs were positive for PPV7. One complete PPV7 strain and eighteen complete *cap* gene sequences were obtained; nucleotide and amino acid identity among the nineteen Cap sequences were 88.1–99.4% and 88.1–100%, respectively. They shared identity with previously discovered sequences ranging from 86.6 to 98.9% and 83.7 to 99.8% at the nucleotide- and amino acid-level, respectively. The phylogenetic tree analysis exhibited that PPV7 strains had two major groups based on the presence or absence of five amino acid (181–185) insertions on the Cap protein. Analysis of the Cap protein demonstrated that PPV7 Cap had significant variability, implying that PPV7 evolved at high substitution rates. Substantial variations of that PPV7 Cap may enable the emergence of newly mutated capsid profiles due to its viral adaptation to host responses. Furthermore, antigenic alteration owing to PPV7 Cap protein amino acid mutations at immune epitopes may enable viruses to escape from the host’s immune system. This study determined the prevalence and genetic characteristics of PPV7 circulating in swine in Hunan, China, and provided the impetus and basis to further investigate the pathogenicity and epidemiology of PPV7.

## 1. Introduction

The family *Parvoviridae* includes two subfamilies: *Parvovirinae* and *Densovirinae*, based on whether these viruses have vertebrates or arthropod hosts, respectively [1]. The subfamily *Parvovirinae* contains viruses that can infect vertebrate hosts, such as birds, cows, pigs, etc. [1]. Porcine parvoviruses (PPVs) belong to the subfamily *Parvovirinae*; to date, seven genotypes of PPVs, designated PPV1–7, have been identified in swine. Currently, PPV includes nine genera, of which *Protoparvovirus* (PPV1), *Teraparovirus* (PPV2 and PPV3), *Bocaparvovirus* (porcine bocavirus), *Copiparvovirus* (PPV4, PPV5, and PPV6), and *Chaphamaparvovirus* (PPV7) infect pigs [2]. PPV7, a small, non-enveloped, single-stranded linear DNA virus (~4 kb genome), was first discovered by next-generation sequencing in rectal swab samples from pigs in the USA in 2016 [3]. The PPV7 genome contains two major open reading frames (ORFs); ORF1 is at the 5′-end and encodes non-structural protein 1 (NS1) involved in viral replication, whereas ORF2 is at the 3′-end and encodes the major structural capsid protein (Cap) or viral protein (VP) [3].

In China, PPV7 has been reported in Guangdong, Anhui, and Guangxi Provinces [4,5,6]. Although the pathogenicity of PPV7 in pigs remains to be elucidated, the high positive rate for PPV7 in aborted pig fetuses and in sows that experienced reproductive failure suggests its association with reproductive failure [7,8]. In addition, co-infection of PPV7 and PCV2 has been reported. The prevalence of PPV7 on PCV2-positive farms was significantly higher than that on PCV2-negative farms, implying that PPV7 may enhance PCV2 infection [4]. In a recent study, a high co-infection rate of PPV7 and PCV2 indicated that PPV7 may act as a co-factor infection involved in PCV2 infection and transmission [6]. In our previous study, we reported that PCV3 viremias were significantly higher in swine that were positive for PPV7 than in those that were negative for PPV7, implying that PPV7 can stimulate PCV3 replication [8]. Therefore, a high prevalence of PPV7 in pigs and co-infections with PCV2 or PCV3 suggest that PPV7 threatens swine herd health security. However, knowledge of the distribution of PPV7 in China is currently limited. Therefore, the purpose of this study was to investigate the prevalence and genetic characterization of PPV7 in Hunan, China.

## 2. Materials and Methods

### 2.1. Sample Collection and Viral DNA Extraction

Four hundred and twenty-two field samples (including lungs, spleens, lymphoid nodes, serum, and semen) from 422 pigs were collected during 2015–2017 from 21 commercial farms in Hunan Province, China. Viral DNA was extracted using the AxyPrep Viral DNA/RNA Miniprep Kit (Axygen Biotechnology, Hangzhou, China) according to the manufacturer’s instructions and stored in a freezer at −20 °C.

### 2.2. PCR Detection and Genomic DNA Amplification

A specific PCR assay to detect PPV7 DNA, with primers targeting a 400 bp segment of *ns1* gene (PPV7-1-F: 5′-AAGAGCGACAAACGCAACAC-3′ and PPV7-1-R: 5′-TGGATCTGCGAACGAACACA-3′). The complete *cap* gene was amplified using a specific primer pair designed according to reference strain PPV7-GX48 (Accession No. MG543469): a forward primer 5′-CTA*GCTAGC*ATGGCAGAACACATCACCCTG-3′ and reverse primer 5′-CCG*GAATTC*TTATTTTCGGCTGGTTGTTG-3′, as described in [6]. The reaction was carried out in a final volume of 20 μL of mixture containing 10 μL of Premix Taq (Tsingke, Beijing, China), 2 μL of the extracted DNA, 1 μL of 10 μM forward primer, 1 μL of 10 μM reverse primer, and 6 μL of ddH_2_O. The PCR conditions were as follows: pre-denaturation for 5 min at 95 °C, followed by 35 cycles of 30 s at 94 °C, 30 s at 60 °C, an extension for 50 s at 72 °C, and a final extension for 7 min at 72 °C. The PCR products were purified using a Gel Extraction kit (Omega, Beijing, China) and inserted into a pCI-neo Vector (Promega, Madison, WI, USA) according to the manufacturer’s instructions. The plasmids were sent for DNA sequencing using a universal primer. Complete PPV7 genome sequence amplification was performed using viral metagenomic deep sequencing with a MiSeq Reagent Kit (Illumina, San Diego, CA, USA).

### 2.3. Alignment and Phylogenetic Analysis

For the alignment of multiple sequences, 19 complete PPV7 Cap genome sequences derived from this study and 64 reference sequences collected from NCBI were analyzed. Multiple sequence alignments and sequence identities were analyzed with the Clustal W method of the MegAlign program of DNASTAR, version 7.10 (Lasergene, Madison, WI, USA). A neighbor joining (NJ) tree was reconstructed using a p-distance model with 1000 bootstrap replicates, and the maximum likelihood (ML) tree was reconstructed using the Jones–Taylor–Thornton (JTT) model of MEGA 7.0 with 1000 bootstrap replicates. Amino acid sequence variations were analyzed using the Virus Pathogen Database and Analysis Resource (ViPR) analysis tool (https://www.viprbrc.org/, accessed on 30 July 2024).

## 3. Results

### 3.1. Detection of PPV7 and Sequences Amplification

**Figure 1:**
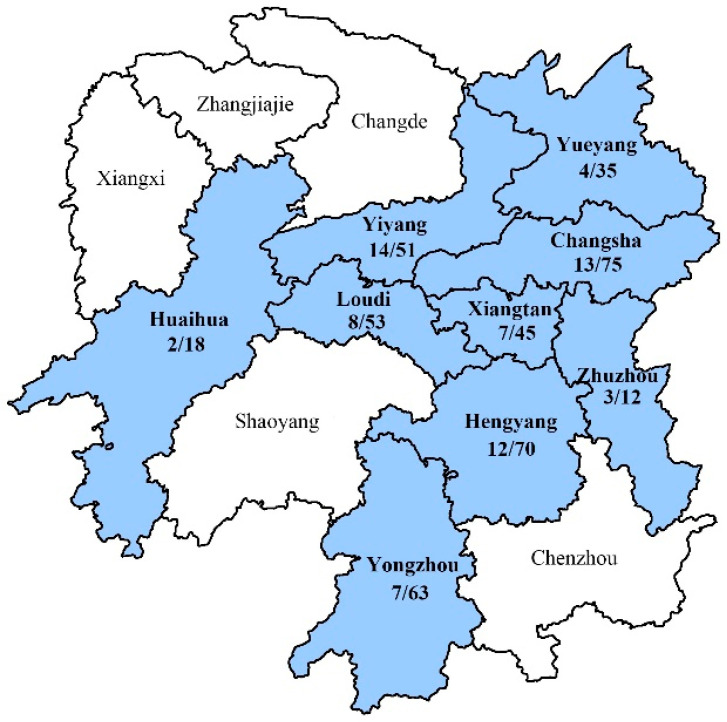
Geographical distribution of PPV7 in Hunan Province. Blue regions represent locations of swine with samples that were positive for PPV7.

Based on the PCR results, PPV7 was detected in swine on farms near nine cities in Hunan Province (Figure 1). The positive rate of PPV7 in these samples was 16.6% (70/422). Specifically, PPV7 was detected in tissues (lung, spleen, and lymphoid node), serum, and semen samples. Of these, 20.2% (58/287) of tissue samples, 15.6% (7/45) of the sera, and 5.6% (5/90) of semen were positive. All positive samples were subjected to genomic amplification. In total, one complete PPV7 genome sequence and 18 *cap* gene sequences were obtained and submitted to GenBank (accession numbers MZ803089-MZ803107, Table S1).

### 3.2. Homology and Phylogenetic Analyses of PPV7 Cap Sequences

The complete PPV7 stain, named PPV7-YiY-25 (accession number: MZ803107), had 95.44% identity to isolate PPV7-JX38. Nucleotide and amino acid identities among the 19 Cap sequences were 88.1–99.4% and 88.1–100%, respectively. In addition, the 19 Cap sequences identified in this study shared ranges from 86.6 to 98.9% and from 83.7 to 99.8% compared to reference strains at the nucleotide and amino acid levels, respectively. In comparative analyses on the PPV7 Cap, 15 *cap* genes identified in this study were 1425 bp in nucleotide length due to successive five amino acid (181–185 aa) insertions, whereas isolates PPV7-YiY-25 and PPV7-YZ-44 were 1416 bp, due to 2 amino acid (181–182 aa) insertions into the Cap protein (Figure S1). In addition, the nucleotide length of one isolate (PPV7-YZ-46) was 1401 bp, due to six amino acid (143–148 aa) deletions and concurrently an insertion of three amino acids (181–183 aa) into the Cap protein (Figure S1). In contrast, only one isolate (PPV7-YiY-30-1) did not have the insertion with a length of 1410 bp (Figure S1).

**Figure 2:**
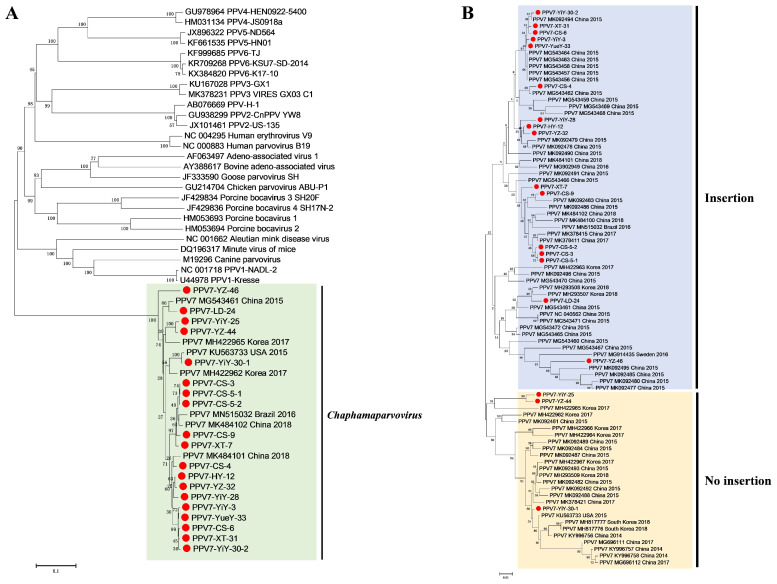
Phylogenetic trees of the *Parvovirinae* subfamily and PPV7 strains. The phylogenetic trees were reconstructed based on Cap amino acid sequences using p-distance-based neighbor-joining (NJ) method (**A**) and using the Maximum-likelihood (ML) method (**B**) with Jones-Taylor-Thornton (JTT) model of MEGA 7.0 with 1000 bootstrap replicates, respectively. Red solid circles indicate the strains obtained in this study.

The neighbor joining (NJ) tree showed that all 19 isolates identified in this study were in the same branch as PPV7 strains belonging to the *Chaphamaparvovirus* genus (Figure 2A). The maximum likelihood (ML) tree was reconstructed with 83 Cap amino acid sequences exhibited that PPV7 strains had two major groups based on the presence or absence of five amino acid insertions (181–185) on the Cap protein (Figure 2B).

### 3.3. Analysis of Cap Amino Acid Sequences

**Figure 3:**
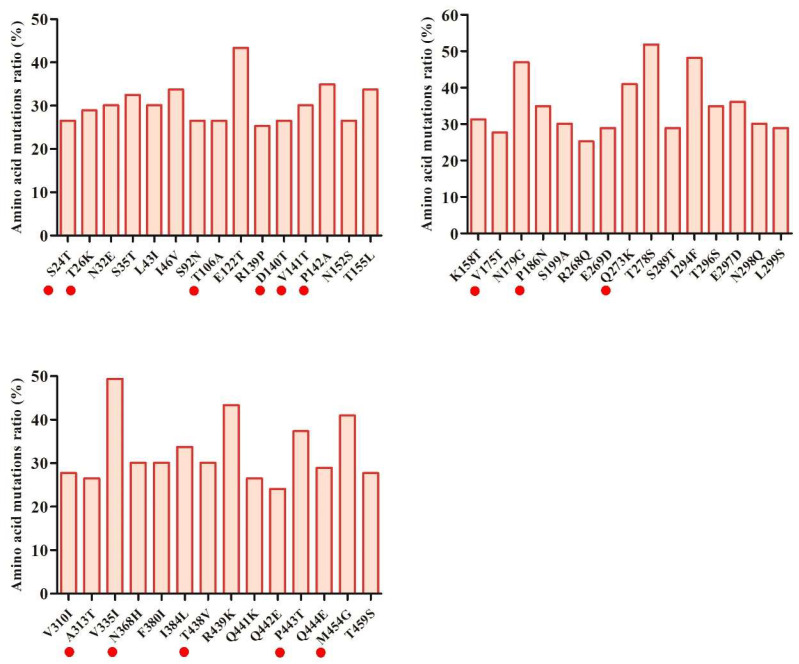
Ratio of high-frequency amino acid mutations in the Cap protein of all PPV7 isolates. Red solid circles indicate ratio of high-frequency amino acid mutations in Cap protein in our PPV7 isolates obtained in this study.

To investigate Cap amino acid variations amongst PPV7 isolates, alignment of Cap protein was performed within 19 isolates in our study and 64 reference PPV7 strains collected from the NCBI database (Table S1). In total, 174 amino acid mutations were detected among Cap proteins (Table S2). The majority of variant PPV7 strains had 44 high-frequency amino acid mutations (Figure 3). Moreover, 14 high-frequency mutations on Cap occurred in these amino acid positions in our isolates (Table 1, Figure 3). Notably, most of these high-frequency mutations occurred predominantly in loops and in predicted potential linear B cell immune epitopes that we had reported [9]. Interestingly, one mutation in isolate PPV7-XT-7 (V136A) significantly increased the antigenic score from 0.4355 to 0.7372 in a predicted B cell epitope (^135^L**V**PKPTTATKEGVGNS^150^ changed to ^135^L**A**PKPTTATKEGVGNS^150^). However, one mutation (T113S) decreased the antigenicity score from 0.5888 to 0.3919 in the predicted linear B cell epitope (^111^YE**T**GYHNW^118^ changed to ^111^YE**S**GYHNW^118^) [9].

## 4. Discussion

Since the discovery of PPV7 in fecal swabs from pigs in the USA in 2016 [3], PPV7 has been subsequently detected in South Korea, Sweden, Poland, and Brazil [7,10,11,12,13]. Moreover, PPV7 has become prevalent since 2014 in Guangdong, Anhui, and Guangxi Provinces in China [4,5,6]. The positive rate of PPV7 was 74% (148/200) in Anhui, 27.3% (105/385) in Guangxi, and 15.4% (67/435) for samples collected from the Hebei, Xinjiang, Shandong, Henan, Jiangsu, Fujian, and Guangdong Provinces [14,15]. In the present study, 287 tissue samples, 45 serum samples, and 90 semen samples were collected from 422 pigs for the epidemiological investigation of PPV7 in Hunan Province, China. Overall, 16.6% (70/422) of the samples had a positive rate for PPV7, and the virus was detected in the lung, spleen, lymphoid node, serum, and semen. Therefore, we inferred that PPV7 is commonly distributed in pigs and may have a wide tissue tropism. Notably, based on detection of PPV7 in semen, this virus may cause reproductive dysfunction via vertical transmission, which supports its prevalence in aborted pig fetuses and in sows that experienced reproductive failure [7,8], suggesting its association with reproductive failure. Moreover, the coinfections of PPV7 and PCV2 or PPV7 and PCV3 have been investigated. The coinfection rate of PPV7 and PCV2 was 17.5% (21/20) in Anhui and 17.4% (65/385) in Guangxi [5,6], while the coinfection rate of PPV7 and PCV3 was 9.1% (11/120) in Anhui [5] and 31.4% (33/105) in our previous study. These results indicate that PPV7 may potentially act as a co-factor involved in PCV2 or PCV3 infection and transmission in pigs. However, the main limitation of this study is that the collected samples focus only on Hunan Province. To fully understand the epidemiology and genetic characterization of PPV7 in China, we will focus on the virus’s evolutionary dynamics based on clinical samples obtained from several regions in China.

To investigate the genetic diversity of PPV7, 19 Cap genome sequences were sequenced, increasing our understanding of the genetic variation and evolution of PPV7 strains. The PPV Cap protein comprises major antigenic domains, making the Cap an ideal candidate immunogen that plays a vital role in inducing neutralizing antibodies [16]. Thus, it is necessary to consider amino acid mutations in the PPV7 Cap protein during virus evolution. Based on our analyses of 83 PPV7 Cap proteins, high-frequency mutations occurred in 44 amino acids, whereas 14 high-frequency mutations occurred in our 19 isolates, predominantly in loops. Furthermore, the Cap protein is the sole structural protein of PPV7, and numerous variations in the exposed surface loops may affect receptor binding [9]. The diversity of variations in the PPV7 *cap* gene was further emphasized by the fact that the virus was evolving at high substitution rates (10^−3^ per site per year), as noted in our previous study [9]. Therefore, it was hypothesized that numerous variations in the PPV7 Cap may lead to the emergence of newly mutated capsid profiles due to viral adaptation to the host. Antigenic alteration owing to PPV7 Cap protein amino acid mutations at immune epitopes may enable the virus to escape from the host’s immune system.

In conclusion, the present study confirmed the prevalence of PPV7 circulating in Hunan Province and included genome sequencing of these PPV7 isolates. Furthermore, the PPV7 Cap had substantial variability, implying that PPV7 was evolving at high substitution rates. Further studies, such as epidemiology, molecular characterization, and genetic diversity, are warranted to elucidate the pathogenesis of this newly emerging PPV7.

## Figures and Tables

**Table 1:** Variable amino acid residues in the Cap protein among 19 PPV7 isolates and six reference strains.

Position	Current Isolates (19)	Reference Strains (6)	Position	Current Isolates (19)	Reference Strains (6)
9	3S/16N	2S/4N	200	1A/1E/17G	2A/4G
10	1A/18T	T	201	1P/18T	T
21	1T/18Q	1N/5Q	204	1R/18V	V
**24**	**1T/1P/5A/12S**	**1P/2A/3S**	215	1L/18F	F
**26**	**2K/3V/14T**	**2V/4T**	216	1C/18T	T
29	Q	1R/5Q	230	A	1T/5A
30	2R/17K	K	266	5S/14N	2S/4N
35	3T/16S	1T/5S	268	8Q/11R	1K/2R/3Q
43	3I/16L	1I/5L	270	1R/2Q/16K	1G/5K
92	9N/10S	2N/4S	**273**	**1R/9Q/9K**	**2Q/4K**
**106**	**2A/8F/9T**	**2A/4T**	278	6T/13S	1T/5S
110	4I/15L	2I/4L	284	1Y/18N	N
113	7S/18T	T	287	2N/17H	1Q/5H
122	4T/15E	1T/5E	289	S	1T/5S
126	1Q/18S	S	294	5I/14F	3I/3F
136	1A/18V	V	296	3S/16T	3S/3T
**140**	**1T/1S/8N/9D**	**2N/4D**	299	1S/1K/17L	1N/1S/4L
**141**	**1N/3T/15V**	**V**	**313**	**1L/8T/10A**	**3T/3A**
**142**	**1T/2Q/3A/13P**	**1T/5P**	327	2K/17R	R
155	6L/13T	3L/3T	**368**	**1K/9N/9H**	**1H/5N**
158	1T/3R/15K	2R/4K	371	1I/18L	L
163	1T/18A	1T/5A	380	9I/10F	1I/5F
165	2A/17I	1A/5I	384	9I/10L	1L/5I
**175**	**1T/1I/4G/13V**	**2G/4V**	434	3K/16R	R
178	T	1P/5T	**438**	**1D/2A/6V/10T**	**1M/2T/3V**
179	5N/14G	2N/4G	439	8R/11K	1K/5R
180	3G/16K	1T/5K	441	2K/17Q	1K/5Q
181	1V/17G	G	442	Q	1E/2H/3Q
182	1T/6E/9T	1P/2T/3E	**443**	**1A/9T/9P**	**1T/2P/3Q**
183	1K/15G	1E/2K/3G	444	5E/14Q	2H/4Q
184	A	1I/5A	446	5S/14P	2S/4P
185	1T/2A/14Q	3T/3Q	**454**	**2K/3M/5E/9G**	**3M/3G**
**186**	**1G/1P/6A/11N**	**1T/2N/3A**	455	1E/18T	T
189	T	1P/5T	467	1T/18Y	Y
195	P	1A/5P	470	1P/18T	T
199	S	1A/5S	472	1I/18S	S

Bold indicates amino acid residues with high-frequency mutations. PPV7 reference strains: GenBank accession numbers MN515032, MH422962, MK484101, MK484102, MG543461, and MH422965.

## Data Availability

Data are available on request.

## Supplementary Materials

The following supporting information can be downloaded at: https://www.mdpi.com/article/10.3390/ani14152222/s1, Figure S1: Sequence alignment of PPV7 Cap protein; Table S1: Detailed information of the 19 complete Cap coding sequences obtained in this study and 64 reference sequences; Table S2: Mutated amino acid residues in the Cap protein of 83 PPV7 isolates.
