# Supplementary material for: Epidemiology and Genetic Characterization of Porcine Parvovirus 7 Recovered from Swine in Hunan, China
Source: Animals (Basel). 2024 Jul 31;14(15):2222. doi: 10.3390/ani14152222 (PMC11311032; doi:10.3390/ani14152222)
Supplement: Supplementary file 1 [file animals-14-02222-s001.zip › animals-3105340-supplementary.pdf]

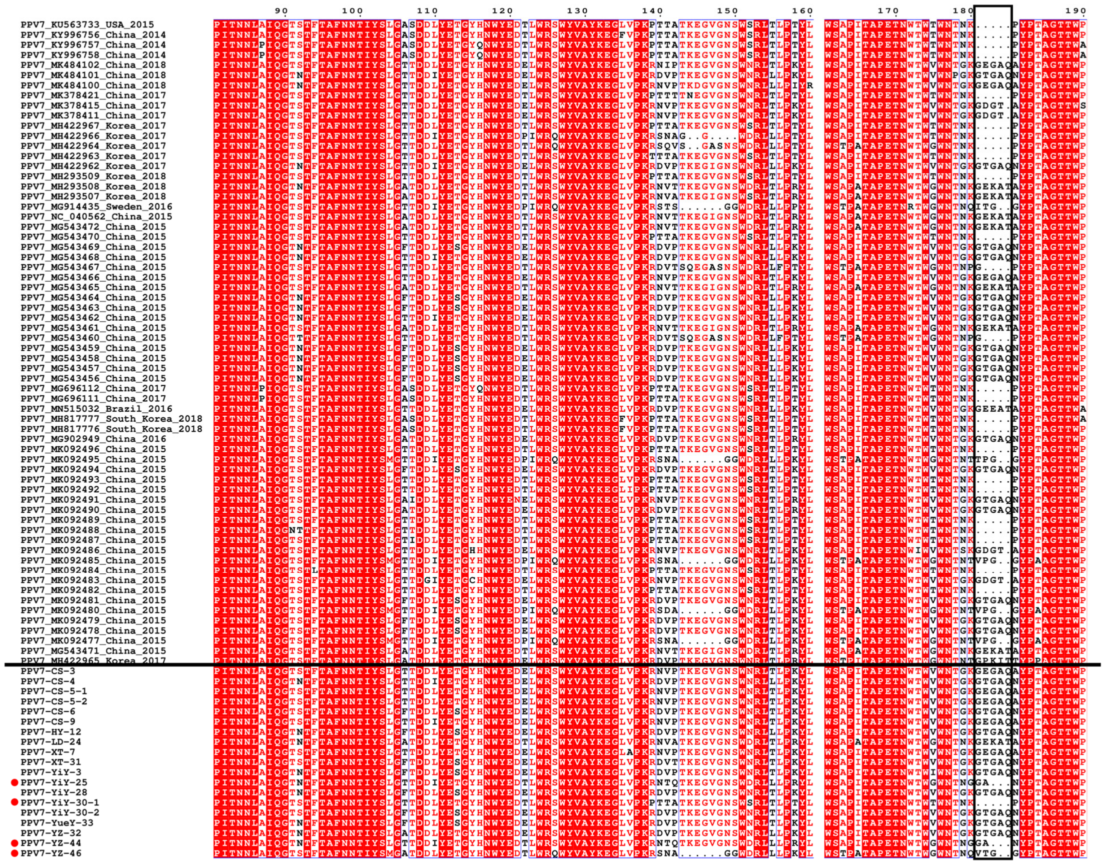

Figure S1. Sequence alignment of PPV7 Cap protein. The successive insertion of five amino acids (181-185 aa) were labelled with black rectangle.

Table S1 Detailed information of the 19 Cap complete coding sequences obtained in this study and 64 reference sequences

GenBank number	Isolate name	Geographic location	Host/Isolation source	Collection date	Length (nt)	Amino acid (aa)
MZ803089	PPV7-CS-3	China (Hunan-Changsha)	Swine/Lung	2017	1425	474
MZ803090	PPV7-CS-4	China (Hunan-Changsha)	Swine/Lung	2017	1425	474
MZ803091	PPV7-CS-5-1	China (Hunan-Changsha)	Swine/Spleen	2017	1425	474
MZ803092	PPV7-CS-5-2	China (Hunan-Changsha)	Swine/Spleen	2017	1425	474
MZ803093	PPV7-CS-6	China (Hunan-Changsha)	Swine/Lung	2017	1425	474
MZ803094	PPV7-CS-9	China (Hunan-Changsha)	Swine/Lung	2017	1425	474
MZ803095	PPV7-HY-12	China (Hunan-Hengyang)	Swine/Lung	2017	1425	474
MZ803096	PPV7-LD-24	China (Hunan-Loudi)	Swine/Lung	2017	1425	474
MZ803097	PPV7-XT-7	China (Hunan-Xiangtan)	Swine/Lung	2017	1425	474
MZ803098	PPV7-XT-31	China (Hunan-Xiangtan)	Swine/Lung	2017	1425	474
MZ803099	PPV7-YiY-3	China (Hunan-Yiyang)	Swine/Lung	2017	1425	474
MZ803100	PPV7-YiY-28	China (Hunan-Yiyang)	Swine/Lung	2017	1425	474
MZ803101	PPV7-YiY-30-1	China (Hunan-Yiyang)	Swine/Lung	2017	1410	469
MZ803102	PPV7-YiY-30-2	China (Hunan-Yiyang)	Swine/Lung	2017	1425	474
MZ803103	PPV7-YueY-33	China (Hunan-Yueyang)	Swine/Lung	2016	1425	474
MZ803104	PPV7-YZ-32	China (Hunan-Yongzhong)	Swine/Serum	2016	1425	474
MZ803105	PPV7-YZ-44	China (Hunan-Yongzhong)	Swine/Serum	2016	1416	471
MZ803106	PPV7-YZ-46	China (Hunan-Yongzhong)	Swine/Serum	2016	1401	466

MZ803107	PPV7-YiY-25	China (Hunan-Yiyang)	Swine/Lung	2017	1416	471
MN515032	BR_RSPPV7	Brazil	Swine	2016	1425	474
MK484102	PPV7/China/AHhf	China	Swine	2018	1425	474
MK484101	PPV7/China/AHmas	China	Swine	2018	1425	474
MK484100	PPV7/China/AHbz	China	Swine	2018	1425	474
MK378411	PPV7_VIRES_GZ02_C1	China	Sus scrofa	2017	1425	474
MG543471	GX49	China	Swine	2015	1425	474
MG543472	GX50	China	Swine	2015	1425	474
MG543470	GX47	China	Swine	2015	1410	469
MG543469	GX48	China	Swine	2015	1425	474
MG543468	GX45	China	Swine	2015	1425	474
MG543467	GX44	China	Swine	2015	1413	470
MG543466	GX35	China	Swine	2015	1425	474
MG543465	GX34	China	Swine	2015	1425	474
MG543464	GX32	China	Swine	2015	1425	474
MG543463	GX31	China	Swine	2015	1425	474
MG543462	GX30	China	Swine	2015	1425	474
MG543461	GX29	China	Swine	2015	1425	474
MG543460	GX28	China	Swine	2015	1413	470
MG543459	GX6	China	Swine	2015	1425	474

MG543458	GX5	China	Swine	2015	1425	474
MG543457	GX3	China	Swine	2015	1425	474
MG543456	GX2	China	Swine	2015	1425	474
MG696112	FJLY2017	China	Swine	2017	1401	466
MG696111	FJFZ2017	China	Swine	2017	1401	466
KY996756	GD-2014-1	China	Swine	2014	1410	469
KY996757	GD-2014-2	China	Swine	2014	1401	466
KY996758	GD-2014-3	China	Swine	2014	1401	466
MG902949	37	China	Sus scrofa/tonsil	2016	1425	474
MK092496	PPV7-JX38	China	Wild boar	2015	1410	469
MK092495	PPV7-JX21	China	Wild boar	2015	1401	466
MK092494	PPV7-JX15	China	Wild boar	2015	1425	474
MK092493	PPV7-JX10	China	Wild boar	2015	1401	466
MK092492	PPV7-DJH26	China	Wild boar	2015	1410	469
MK092491	PPV7-DJH24	China	Wild boar	2015	1425	474
MK092490	PPV7-DJH23	China	Wild boar	2015	1425	474
MK092489	PPV7-DJH20	China	Wild boar	2015	1410	469
MK092488	PPV7-DJH19	China	Wild boar	2015	1410	469
MK092487	PPV7-DJH14	China	Wild boar	2015	1410	469
MK092486	PPV7-DJH13	China	Wild boar	2015	1422	473

MK092485	PPV7-DJH12	China	Wild boar	2015	1401	466
MK092484	PPV7-DJH11	China	Wild boar	2015	1410	469
MK092483	PPV7-LB4	China	Wild boar	2015	1422	473
MK092482	PPV7-87-clone2	China	Wild boar	2015	1410	469
MK092481	PPV7-87-clone1	China	Wild boar	2015	1425	474
MK092480	PPV7-80	China	Wild boar	2015	1401	466
MK092479	PPV7-77	China	Wild boar	2015	1425	474
MK092478	PPV7-60	China	Wild boar	2015	1425	474
MK092477	PPV7-55	China	Wild boar	2015	1401	466
NC_040562	GX49	China	Swine	2015	1425	474
MK378421	PPV7_VIRES_HuN01_C2	China	Sus scrofa	2017	1410	469
MK378415	PPV7_VIRES_GZ04_C2	China	Sus scrofa	2017	1422	473
MH422967	PPV7-KF6	Korea	Pig	2017	1410	469
MH422966	PPV7-KF5	Korea	Pig	2017	1392	463
MH422965	PPV7-KF4	Korea	Pig	2017	1425	474
MH422964	PPV7-KF3	Korea	Pig	2017	1404	467
MH422963	PPV7-KF2	Korea	Pig	2017	1410	469
MH422962	PPV7-KF1	Korea	Pig	2017	1425	474
MH293509	PPV7-KA3	Korea	Pig	2018	1410	469
MH293508	PPV7-KA2	Korea	Pig	2018	1425	474

MH293507	PPV7-KA1	Korea	Pig	2018	1425	474
MH817777	N133	South Korea	Pig	2018	1410	469
MH817776	N141	South Korea	Pig	2018	1410	469
MG914435	PPV7_SWE20	Sweden	Pig	2016	1401	466
KU563733	42	USA	Swine	2015	1410	469

Table S2 Mutated amino acid residues in the capsid protein of 83 isolates

Positions	Amino acid	Mutations (Number of variant strains)		
5	I	T (1)		
8	S	N (1)		
9	N	S (9)		
10	T	A (1)		
18	D	A (4)		
21	Q	T (6)	N (2)	
24	S	T (22)	A (13)	P (10)
25	Y	H (1)		
26	T	K (24)	V (19)	
29	Q	R (1)		
30	K	R (10)		
32	N	E (25)	D (1)	K (1)
33	V	I (3)		
35	S	T (27)	P (1)	
36	Y	S (4)		
37	N	S (3)		
42	I	V (8)		
43	L	I (25)		
44	P	L (4)		
46	I	V (28)		
70	E	K (1)		
76	V	I (3)		
87	A	P (4)		
91	T	N (1)		
92	S	N (22)	T (2)	
94	F	L (1)		
104	L	M (5)		
106	T	A (22)	F (18)	
107	T	S (8)	I (2)	
109	D	G (1)		
110	L	I (15)		
113	T	S (18)		
115	Y	C (1)	H (1)	
116	H	Q (3)		
121	D	N (1)		
122	E	T (36)	P (6)	
123	L	I (6)		
126	S	Q (8)		
135	L	F (3)		
136	V	A (1)	I (1)	
139	R	P (21)	T (1)	

140	D	T (22)	N (23)	S (8)		
141	V	T (25)	N (5)	D (1)	Q (1)	I (1)
142	P	A (29)	T (10)	Q (2)	S (1)	V (1)
143	T	S (3)	G (1)			
144	K	Q (2)	N (1)			
145	E	D (1)				
147	V	I (6)	A (3)			
148	G	S (3)				
149	N	G (6)				
150	S	G (6)				
152	N	S (22)	D (19)			
155	T	L (28)				
156	L	F (2)				
158	K	T (26)	R (14)	P (1)	I (1)	
160	L	R (1)				
163	A	T (10)				
165	I	A (17)				
172	W	R (1)				
173	T	I (1)				
175	V	T (23)	G (20)	I (1)		
178	T	P (1)				
179	N	G (39)	S (1)			
180	K	T (5)	Q (2)	G (2)	P (2)	
181	G	V (4)	I (1)	T (1)		
182	T	E (17)	P (5)	D (4)	A (2)	
183	G	K (9)	E (1)			
184	A	T (4)	I (1)			
185	Q	T (10)				
186	P	N (29)	A (21)	G (6)	T (1)	
189	T	A (3)	P (1)			
195	P	A (4)	S (1)			
199	S	A (25)	P (1)			
200	G	A (11)	D (3)	E (2)	T (1)	
201	T	P (6)				
204	V	R (11)				
205	A	V (4)				
209	G	R (4)				
211	F	S (1)				
215	F	L (14)				
216	T	C (5)				
218	P	T (4)	L (1)			
225	R	M (1)				
227	G	R (1)				

230	A	T (1)						
231	M	V (1)						
232	S	G (2)						
234	H	R (1)						
236	K	N (3)						
239	G	A (4)						
246	Y	H (1)						
248	L	P (1)						
250	S	R (2)						
266	N	S (10)						
268	R	Q (21)	K (1)					
269	E	D (24)	N (23)	G (1)				
270	K	G (11)	Q (2)	R (1)				
271	K	T (3)	Q (1)					
273	Q	K (34)	R (1)					
277	G	E (4)						
278	T	S (43)						
280	I	V (17)						
284	N	Y (4)						
287	H	Q (6)	N (3)	R (1)				
289	S	T (24)						
291	Q	R (1)						
294	I	F (40)						
296	T	S (29)						
297	E	D (30)						
298	N	Q (25)						
299	L	S (24)	Q (3)	P (2)	H (2)	K (1)	N (1)	R (1)
300	K	N (1)						
302	F	L (2)						
304	E	Q (4)						
307	V	I (2)						
310	V	I (23)						
312	N	T (4)						
313	A	T (22)	L (1)					
315	I	S (4)						
318	I	V (1)						
319	Q	H (4)						
327	R	K (7)						
328	N	S (3)						
335	V	I (41)						
340	L	M (2)						
344	G	D (1)						
349	T	I (1)						

357	N	D (1)			
358	F	V (1)			
359	I	T (1)			
360	K	Q (1)			
362	I	V (15)			
368	N	H (25)	K (10)		
369	G	E (4)			
371	L	I (6)			
373	K	N (1)			
375	V	I (6)			
376	T	A (1)			
379	C	Y (1)			
380	F	I (25)	Y (3)		
383	S	G (4)			
384	I	L (28)			
387	S	A (5)			
406	E	Q (2)			
411	I	T (1)			
412	D	N (7)			
413	S	R (4)			
419	T	P (4)			
421	R	G (1)			
429	R	G (1)			
434	R	K (17)			
437	D	G (1)			
438	T	V (25)	A (13)	M (1)	
439	R	K (36)	Q (4)		
441	Q	K (22)	G (6)		
442	Q	E (20)	H (3)		
443	P	T (31)	Q (11)	A (2)	S (1)
444	Q	E (24)	H (3)		
445	Q	K (6)	H (2)		
446	P	S (11)	T (9)	R (3)	G (1)
448	Y	D (1)			
449	Q	E (1)			
453	Y	C (1)			
454	M	G (34)	E (13)	K (8)	
455	T	E (1)			
457	T	A (1)			
459	T	S (23)			
461	T	A (1)			
462	T	A (3)			
465	S	N (1)			

467	Y	T (1)	
470	T	I (3)	P (1)
472	S	I (1)	
473	R	Q (1)	
